# DB4US: A Decision Support System for Laboratory Information Management

**DOI:** 10.2196/ijmr.2126

**Published:** 2012-11-14

**Authors:** José M Carmona-Cejudo, Maria Luisa Hortas, Manuel Baena-García, Jorge Lana-Linati, Carlos González, Maximino Redondo, Rafael Morales-Bueno

**Affiliations:** 1 University of MalagaComputer Sciences and Languages DepartmentMálagaSpain; 2 Hospital Costa del SolClinical Laboratories DepartmentMarbellaSpain; 3 Clínicas RincónRincón de la VictoriaSpain; 4 Siemens Healthcare DiagnosticsBarcelonaSpain; 5 Laboratorios Clínicos, REDISSECMarbellaSpain

**Keywords:** Automation, laboratory, Medical Informatics Applications, Data Mining, Quality Indicators, Health Care

## Abstract

**Background:**

Until recently, laboratory automation has focused primarily on improving hardware. Future advances are concentrated on intelligent software since laboratories performing clinical diagnostic testing require improved information systems to address their data processing needs. In this paper, we propose DB4US, an application that automates information related to laboratory quality indicators information. Currently, there is a lack of ready-to-use management quality measures. This application addresses this deficiency through the extraction, consolidation, statistical analysis, and visualization of data related to the use of demographics, reagents, and turn-around times. The design and implementation issues, as well as the technologies used for the implementation of this system, are discussed in this paper.

**Objective:**

To develop a general methodology that integrates the computation of ready-to-use management quality measures and a dashboard to easily analyze the overall performance of a laboratory, as well as automatically detect anomalies or errors. The novelty of our approach lies in the application of integrated web-based dashboards as an information management system in hospital laboratories.

**Methods:**

We propose a new methodology for laboratory information management based on the extraction, consolidation, statistical analysis, and visualization of data related to demographics, reagents, and turn-around times, offering a dashboard-like user web interface to the laboratory manager. The methodology comprises a unified data warehouse that stores and consolidates multidimensional data from different data sources. The methodology is illustrated through the implementation and validation of DB4US, a novel web application based on this methodology that constructs an interface to obtain ready-to-use indicators, and offers the possibility to drill down from high-level metrics to more detailed summaries. The offered indicators are calculated beforehand so that they are ready to use when the user needs them. The design is based on a set of different parallel processes to precalculate indicators. The application displays information related to tests, requests, samples, and turn-around times. The dashboard is designed to show the set of indicators on a single screen.

**Results:**

DB4US was deployed for the first time in the Hospital Costa del Sol in 2008. In our evaluation we show the positive impact of this methodology for laboratory professionals, since the use of our application has reduced the time needed for the elaboration of the different statistical indicators and has also provided information that has been used to optimize the usage of laboratory resources by the discovery of anomalies in the indicators. DB4US users benefit from Internet-based communication of results, since this information is available from any computer without having to install any additional software.

**Conclusions:**

The proposed methodology and the accompanying web application, DB4US, automates the processing of information related to laboratory quality indicators and offers a novel approach for managing laboratory-related information, benefiting from an Internet-based communication mechanism. The application of this methodology has been shown to improve the usage of time, as well as other laboratory resources.

## Introduction

These days, laboratories tend towards automation. The data they manage are structured around information systems that store not only purely analytical data, but also management data, such as samples, timestamps, and demographics. Nevertheless, there is a lack of ready-to-use management quality measures or indicators, which makes it very difficult to easily analyze the overall performance of the laboratory and thus to use the available information to make evidence-supported decisions [1].

The reagents used for laboratory analysis have a profound impact on the global economic balance of the laboratory. Clinical laboratory costs increase rapidly due to increased laboratory utilization and to inflationary trends [2]. In particular, the Hospital Costa del Sol laboratory expenditures increase about 10%-15% annually. The costs of this laboratory have an impact of 3-4% on the total hospital expenditures.

It has been pointed out that having accurate information on laboratory test costs effectively leads to reduction in hospital expenditure [3]. For that reason, an accurate estimation of the money spent on reagents in a given period is of great importance from an organizational point of view. In order for it to be reasonably accurate, a large amount of data on number of determinations, quality controls, or calibrations for different tests and analytical instruments has to be available beforehand. These data are already stored in the instruments and information systems of the laboratory, but not in an easily accessible fashion. Each data source stores a large amount of partially redundant data using a different format. The collection and analysis of all the necessary data represents a cumbersome, error-prone, and time-intensive process, and the estimation of the desired values has to make use of extrapolation of past trends and other indirect means. Such calculations involve several hours, or even days, of manual work, combining data from different sources.

Furthermore, interesting information such as patterns and trends can be hidden among the massive amounts of data distributed between different systems. For this reason, it would be of great use to have a system able to gain access to all the necessary data in the information systems, in order to automatically unify, process, and provide ready-to-use summaries to the laboratory manager. Indicators defined by the user would be readily available, allowing relevant performance trends to be seen at a glance and possibly revealing some aspects of the laboratory performance that could be optimized [4].

Decision support systems capable of providing such performance measures constitute a solution for this task, as explained by Power’s article [5], which offers a review of the most important milestones in the history of decision support systems. In that work, decision support systems are presented as the result of the convergence of various technology threads, which started in the early 1960s. The focus shifted towards web-based analytical applications in the late 1990s, with the emergence of ad-hoc corporate intranets and web-based applications. The adoption of such information systems has been a key factor for quality improvement in medical centers such as the Legacy Good Samaritan Hospital in Portland, Oregon, the Ranking Medical Center in Brandon, Mississippi, and St. Mary’s Health Care System in Athens, Georgia [6]. Some specific examples of successful decision support systems for clinical laboratories are QCIS [7], designed to identify relevant clinical information, and COAT [8], a system able to assess outcomes and measure performance by gathering, formatting, and abstracting data.

A suitable tool for providing quality measures is the management dashboard, an interface where complex and heterogeneous data from various sources are consolidated and displayed, in order to provide easy-to-read summaries of previously defined performance metrics. Dashboards are the result of the evolution and convergence of classical Decision Support Systems, Executive Information Systems, Data Warehouses, and Business Intelligence [9]. They represent a key tool to improve efficiency, accelerate decisions, and reduce oversights and errors in clinical practice [10]. We refer to the work of Kroch et al [11], where several dashboards across different hospitals are studied, showing the tool’s effectiveness in quality management.

In this paper, we present a methodology based on a management dashboard providing performance measures for laboratories and a web application that illustrates this methodology, DB4US. This tool is the product of a project created as part of an ongoing collaboration between the University of Málaga, Hospital Costa del Sol of Marbella, and Siemens Healthcare Diagnostics, S.L.

Our goal was to design a methodology that would enable physicians to obtain information about the overall performance of the laboratory in the form of requests, tests, and turn-around times and to validate this methodology implementing a web application. As an easy-to-read summary of the data, we propose the use of a dashboard screen, which offers a high-level view of the most important indicators.

The main objective is thus the design of this methodology and the implementation and deployment of a dashboard-like application capable of summarizing metrics related to the number of tests, samples, and requests handled by the laboratory of the Hospital, as well as turn-around times. The implementation of the project comprises the construction of a unified data warehouse that stores and consolidates multidimensional data from various information sources, offering an interface to obtain ready-to-use indicators, and a user-oriented front-end application that offers the possibility to drill down from high-level metrics to more detailed summaries.

## Methods

The laboratory of the Hospital Costa del Sol is organized around the ADVIA LabCell system, with several analytic instruments: two ADVIA 2400, three ADVIA Centaur XP systems, and one IMMULITE 2000 Xpi system. The core of the information system of the laboratory is the ADVIA CentraLink data management system, which provides an integrated foundation for automating and consolidating data from the laboratory. Another important information source in the laboratory is Linemaster, which offers more detailed information about the samples’ life-cycle and their related timestamps.

The Lab Cell System processes around 1200 patients daily and has a portfolio of 97 different tests (eg, biochemistry, immunochemistry, serology, allergy tests, etc.). The impact on the general budget of the laboratory is 50%, approximately. Note that our application uses only data from the automation chain. Data that are not provided by the aforementioned systems are out of the scope of the application described in this article.

It is necessary to access the different sources of data to achieve the objectives, specifically the ADVIA CentraLink system and Linemaster, to consolidate the obtained data and to calculate several statistical indicators to be concise, informative, and easy to read and interpret [12-14].

The methodology followed by DB4US works with up-to-date data, which means that processes have to be designed to regularly extract data from the sources and store that data in a data warehouse. As the application works with large amounts of data, these extraction processes have been carefully designed to be as efficient as possible with respect to execution time, as well as to memory and storage space.

The users of this application cannot retrieve all the detailed information stored in the database, but only summarized views or indicators, such that any given indicator can be immediately retrieved from the data warehouse. For that reason, the offered indicators have been calculated beforehand, so that they are ready to use when the user needs them. This necessity leads to the design of a set of different parallel processes to precalculate indicators, employing the consolidated data from the data warehouse.

A graphical user interface (GUI) offers easy access to the indicators. Its structure makes it clear how to move around the different sections of the statistics. Those are presented in an informative and interpretable way, using information representation techniques such as data charts and tables. For any given indicator, the GUI allows us to compare its value for different months and years. It has been made flexible and customizable, offered in several languages, and the association between instruments, groups of instruments, and parameters, as well as the user-defined thresholds, is configurable.

To provide a more detailed methodological and architectural vision, the description of DB4US is divided into two sections. The first section is devoted to describing detailed information about the specific indicators we are working with, and the other one to describing the architecture and some technical details of the implementation.

### Indicators Description

We have implemented an application that displays three main kinds of information. The first one corresponds to tests, requests, and samples. The user can look up general information about requests and their associated samples. This allows the user to discover the ratio between these two parameters, showing how many requests have resulted in more than one sample. Parameter-based information can be also looked up, organized by instrument groups and specific parameters, and broken down into requested and uploaded tests, repetitions, quality controls, and calibrations. The second kind of information is related to turn-around times [15], showing global and detailed turn-around time curves for the samples, as well as percentiles. The third kind of information is captured in the dashboard which summarizes laboratory status and gives a global view of activity.

#### Tests, Requests, and Samples

The first batch of indicators is related to activity. The number of requests, tests, and samples are shown on a monthly basis, and the total quantity for the last years, as well as the accumulated quantity for the current year, is shown for comparison. The number of requested and reported tests is also shown. This number will be lower than the number of total tests performed because repetitions, quality control, and calibrations are not included. The ratio between requested and total performed tests is another interesting measure, since it allows the user to assess how much extra work has been carried out.

The number of samples can be grouped by sample origin, while the number of tests can be grouped by group of instruments and specific parameters. Tests can be subsequently broken down into uploaded, repeated, quality controls, and calibrations. The ratio between the real and requested tests can also be calculated for each parameter.

From the number of total tests performed, it is possible to obtain information about test-related costs. Test costs can be configured by the user and can be used in combination with the number of determinations in order to obtain the total costs, both overall and by instruments and parameters.

#### Turn-Around Times

The second batch of indicators is related to the turn-around times. One of the key indicators is the turn-around-time for the 50th and 90th percentiles, or for any other pair of percentiles that the user configures. Since turn-around times do not follow a Gaussian distribution, these percentiles are more informative than averages and variances. The percentiles can be grouped by origin since it is illustrative to compare how samples from different origins behave. Turn-around-times can be broken down into pre-analytical and analytical times.

#### Dashboard

The dashboard is the core of our information system. It is designed to show the following indicators on a single screen:

1. Requests progress: Represents the ratio between the requests for a given month and the requests for the previous month.
2. Tests progress: Represents the ratio between the tests for a given month and the requests for the previous month.
3. Tests/requests ratio: Represents the ratio between the real and the requested tests for each month.
4. Not uploaded tests: Represents the number of tests that have been requested, but not uploaded (eg, because they have not been validated yet).
5. Repetitions/uploaded ratio: Ratio of repetitions for each uploaded result. It is thus a measure of extra work.
6. Quality factor: The ratio of quality controls and calibrations is an indicator of how much reagent has been employed to ensure the quality of the analytical system.
7. Quality factor for different areas ADVIA 2400 system, ADVIA Centaur system, and IMMULITE system: Same as above, but grouped by specific instrument groups.
8. Samples/requests ratio. Represents how many samples are associated with each patient request on average.
9. Samples with turn-around time longer than 10 days: These samples are considered to be anomalous and have to be individually checked by the medical staff.
10. Percentiles 50 and 90 of turn-around time and average values.
11. Percentage of requests with a turn-around time longer than 12 hours: These requests are left for the next day; therefore, it can be considered as the ratio of requests that cannot be completed in the same day. A high number of requests finished after more than 12 hours shows that a problem has delayed the workflow (eg, a system failure).

The web application contains a configuration section where the aforementioned parameters can be modified. For example, the user can opt to monitor samples with turn-around times longer than 12 instead of 10 days, percentiles 95 instead of 90, or percentage of requests with a turn-around time longer than 8 instead of 12 hours.

For each indicator, a normal range is defined. It is also configurable by the web application user, who has previously defined a minimum value *v*_min _and a maximum value *v*_max_. The values are shown, month-by-month, using a three-color code: Green, if the value lies between the defined validity range limit for that indicator; Orange, if the value is outside the limits but the distance to the nearest limit is less than (*v*_max -_ *v*_min_)/2; and Red, if the value is outside the limits and the distance to the nearest limit is greater or equal to (*v*_max -_ *v*_min_)/2.

### System Description: Technologies and Architecture

**Figure 1:**
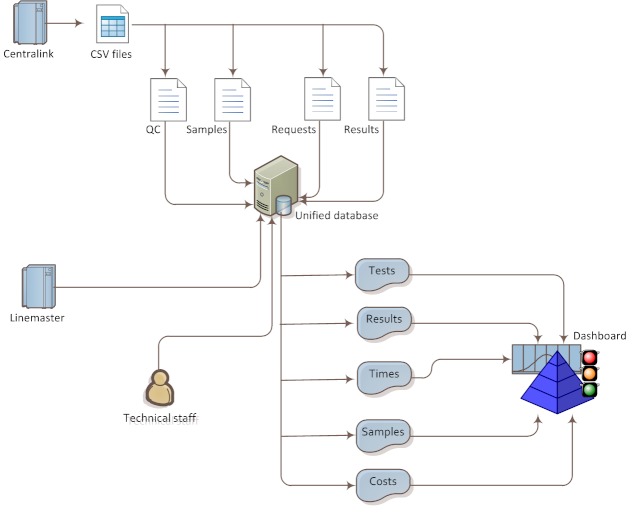
Application structure.

The implemented application is based on Java technologies and uses Java EE-based web architecture, with several layers for data access, transformation, and visualization. An outline of the application structure is shown in Figure 1.

We have chosen to implement an information system from scratch instead of using or adapting an already existing business analytics system such as MicroStrategy Reporting Suite for a number of reasons. Already existing systems are typically based on OLAP technologies, which impose a given structure onto the underlying database. Our application is very intensive in terms of space, so the authors have deemed the extra effort of implementing a database from scratch worth the effort because a balance between space and time needed for queries can be more fine-tuned and adapted to the specific laboratory needs. Furthermore, our business layer provides a data representation that makes the indicators totally independent of the final representation, which helps to reuse data structures between different representation strategies, such as HTML tables, Flash graphics, XML data, or Excel files.

#### Application Architecture

We have implemented our project as a Java EE-based web application [16] that makes use of an external PostgreSQL database [17]. That means that the application has to be installed only once on a server and will be accessible for the final users on their web browsers, without the necessity of installing anything. Java technologies have been chosen because Java is a powerful, platform-independent language with a large number of third party libraries that offer a wide range of functionalities. We have implemented a user interface using the Java Server Faces [18] web framework and InfoSoft Global FusionCharts for graphical charting [19]. The web application relies on Glassfish v.2, an open source application server developed by Sun Microsystems. For the database management system, we have chosen PostgreSQL, an advanced open-source database system. The reasons for the technological decisions are further described in the Discussion section.

The core of our project is the central database, in which clean data from ADVIA CentraLink system and Linemaster is stored, together with several preprocessed summaries, ready to be served to the Java application. Two main database levels have been implemented: the first one stores raw data, which would be used to calculate indicators; the second level is the indicators level, in which the data is summarized.

Our application consists of two main components: a back-end, where calculations take place, and a user-oriented front-end. These two components are further explained in the next subsections.

#### Back-End

The back-end of our application contains modules that directly interact with the central database. This back-end is divided in two main modules. The first one is responsible for consolidating the data originating from the ADVIA CentraLink system and Linemaster databases, as well as from the manually entered data, such as calibrations. The second one is responsible for summarizing the data and for offering ready-to-use indicators to the web application. While the first module is dependent on the specific infrastructure of this laboratory, and thus has to be adapted for each laboratory, the second one is independent and can be used as is in other laboratories.

In order to adapt the first module to the specific infrastructure of the laboratory, the application needs information about which proprietary systems it is connected to and how the database of each of such systems has to be queried in order to obtain information to be analyzed. This is due to the fact that the database structure of each proprietary system is different. This is accomplished by implementing a table that stores certain information (see Table 1).

**Table 1:** Fields in the data table.

Field	Meaning
Query identifier	A unique string identifier for each specific query (eg, number of determinations during the last month)
Proprietary system	The proprietary system to be queried (eg, Advia 2400)
SQL sentence	The SQL query that allows to extract the query defined by the query identifier from the database of the corresponding proprietary system

Additionally, for each proprietary system we need to specify connection parameters (IP address, port, user, and password). These parameters are configurable by the user.

Although the database of each proprietary system is different, it has to be able to provide at least the following parameters in order to be compatible with DB4US: requests; samples associated with each request; demographics associated with each request; tests associated with each sample; parameter, status and result of each test; and timestamps associated with the life-cycle of each test.

**Figure 2:**
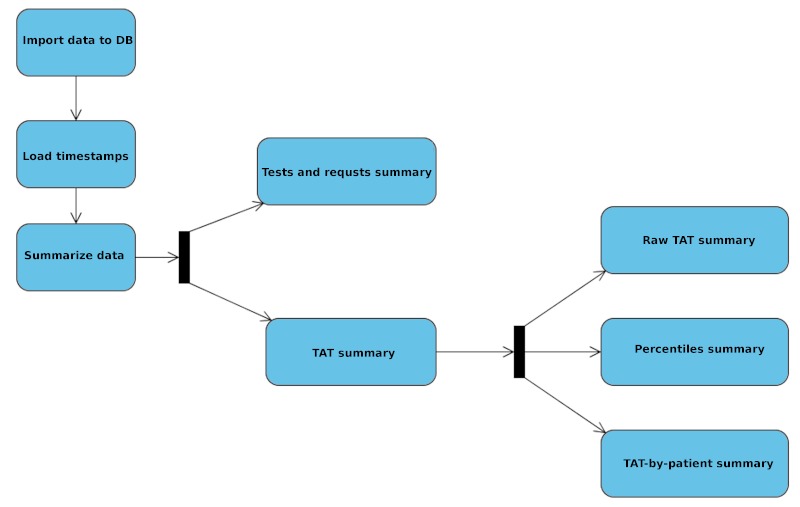
Data loading and summarizing outline.

An important cross-component that affects these two modules is the scheduler, a piece of software that automatically triggers temporized data importations and summaries according to a previously defined temporal planning. We have defined a specific scheduling for this laboratory, but the application lets the user configure new scheduling plans. In our case, the plan runs weekly. Every weekend, data from the ADVIA CentraLink system is exported to CSV files. Once these files are available at the ADVIA CentraLink system main server, our back-end application parses them and loads the data into the central database, previously normalizing the relational data. Simultaneously, time-related data from Linemaster are imported into a different section of the central database. When these two tasks have been successfully completed, several timestamps, which comprise data from both the ADVIA CentraLink system and Linemaster, are computed. The CSV files are then compressed and backed-up. At this point, we have imported all the raw data that we need for building the indicators. For this calculation, several threads are launched simultaneously, each of them specializing in one specific indicator. All of these tasks, as well as the data import, are automatically stored; if the server goes down, the application automatically resumes the pending tasks. See Figure 2 for an outline of the main activities involved.

The back-end application provides another distinct module that serves the indicators. This module waits until an external request for a given indicator is received from another application, such as our web front-end. The necessary chunks of data required for calculating the indicator are retrieved from the database, and the indicator is serialized and returned to the calling application, which usually represents this data as a table or graphic.

#### Front-End

For the front-end, we have implemented another Java EE web-based application that acts as an interface between the back-end and the user. The web application provides such features as customization, security via authentication and authorization modules, internationalization, tabular, and graphical representations of the data, and exportation to other formats, such as Microsoft Excel.

To grant access to the users, a login and password are provided, which give them the permissions associated with a given role. For example, to configure the application, the user has to belong to an administrative role; this is not needed to simply see the data. Once the user has logged in, they can navigate through the application with the help of a menu and links.

**Figure 3:**
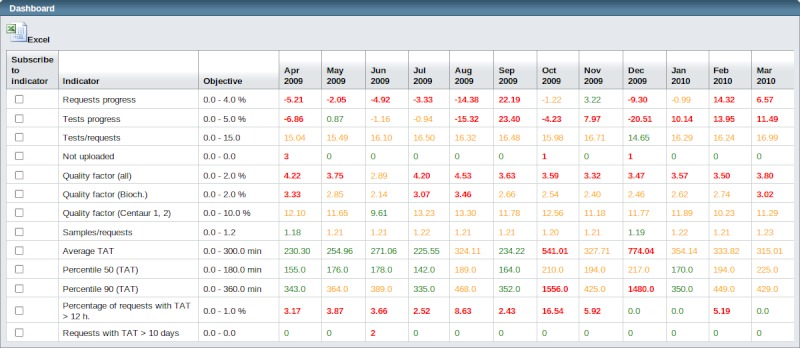
Dashboard screenshot.

The front-end acts interactively. The user’s actions, such as clicks and selections, are transformed into requests to the back-end application, which, in turn, retrieves data from the database. The web application renders the received data into tables and graphics. For the tables, we have used JSF components from the Woodstock library along with an object-oriented representation of their rows. For the graphics, we have implemented a component that transforms the data returned by the back-end application into the XML format. A Servlet (a Java class that responds to http requests with XML code) is associated with each graphic. Along with these main components, we have implemented a number of helper classes for date-related data. We have used Java internationalization components in order to provide a multilingual interface. See Figure 3 for a screenshot of the dashboard.

## Results

DB4US was first deployed in the Hospital Costa del Sol in 2008 and has been in continuous use ever since. The system has been evolving over time, in order to cope with new necessities and ideas. The main benefits of the deployment of the system are, on the one hand, the reduction of the time needed for the calculation of indicators, and on the other, access to a global view of the performance of the laboratory that allows identification of aspects that can be optimized.

The automation of the indicators’ retrieval means that the time delay is heavily reduced. For example, before the deployment of our application, an Excel spreadsheet for calculating the money spent on reagents was manually compiled, with costs and usage data for each type of reagent. This calculation involved several hours of repetitive and error-prone manual work. Thanks to our application, these summaries can be automatically calculated over the weekend, giving the user access to a ready-to-use summary of the data, saving hours of manual work and ensuring error-free data.

Another important advantage of this application is the ability to provide the final user with access to a global perspective of the laboratory performance in terms of reagent usage and turn-around times. This means that the user can easily detect trends at a glance. This new possibility has an additional benefit: if there is something anomalous in the indicators, the user can take action immediately, which gives them a chance to improve some aspects of the process. This was shown in the discovery of the anomalous behavior of the direct bilirubin (BD) test requests. In early 2009, an unlikely high value of the total number of BD requests was spotted in the corresponding indicator provided by our application. The reason for this value turned out to be an incorrectly configured trigger rule in the Centralink system, which triggered an unwanted BD request for any new registered sample. This discovery allowed the laboratory staff to disable this anomalous rule. Therefore, an important fact that was somehow hidden among a vast amount of information was revealed by the automated processing of information carried out by our system. Following this discovery, the number of requests for this rule diminished by an average of 2297.1 requests (taking the previous and succeeding 6 months); ie, a 77.89% less reagent usage for this test type. See Table 2.

**Table 2:** Evolution of direct bilirubin test requests.

Month	Number of requests
August ’08	2255
September ’08	3244
October ’08	3094
November ’08	2729
December ’08	3354
January ’09	2129
February’ 09	621
March ’09	664
April ’09	594
May ’09	634
June ’09	643
July ’09	692

## Discussion

Nowadays, clinical laboratories face numerous challenges such as health care reform, cost pressures, tight laboratory regulations, a growing complexity of diagnostic tests, increased workload, and continuous shortening of turn-around times. At the same time, the users have very high expectations of laboratory services. In order to overcome the above-mentioned obstacles, a clinical laboratory needs to incorporate creative solutions and adapt to change. That is why the introduction of artificial intelligence by means of expert systems has gained an important place in the automation process [20]. To convince our administrators, we need new comprehensive tools for providing indicators of activity and quality. In this work, we aimed to develop a general methodology illustrated by the implementation of an application that integrates the computation of quality measures to easily analyze the overall performance of a laboratory, as well as automatically detect anomalies.

### Principal Results

During the analysis phase of this project, it was decided to deploy the software as a web rather than a desktop application. The use of Internet and web applications has several advantages: no disk space is required by the client, cross-platform compatibility is provided, and when installing new features, only the server side has to be upgraded. Furthermore, the final users do not have to install anything on their computers. The main drawback of this solution is that the application cannot be accessed in case of a network failure.

As for implementing a web application, we had a large number of available technologies to choose from. Two of the most widely used technologies for implementing complex web applications are Microsoft .NET and Java EE. There are several similarities between these two technologies: both run over a virtual-machine environment and both provide a large library of solutions for GUI construction, database access, persistence, caching, or remote method invocation, among others. Java has the advantage of providing cross-platform compatibility and of being open-sourced software. Furthermore, there exist a large number of external Java libraries for data analysis, such as Weka [21]. These reasons have prompted us to adopt Java EE as our main technology.

There are quite a few web application frameworks that work under Java, many of them following the model-view-controller pattern to separate the data model and business rules from user interface. Examples of these frameworks are Apache Struts, Spring and Sun Java Server Faces (JSF), all of which provide features for security, URL mapping, templating, and Ajax support. We have decided to use Sun JSF, which provides Facelets, a simple, efficient, and powerful view description language. Java EE-based web applications run on an application server. For our purposes, we use Oracle Glassfish, which is based on Apache Tomcat.

As one of the key goals of our application is to provide an easily comprehensible view of data, the use of adequate data visualization components is of utmost importance. InfoSoft Global FusionCharts is a library to automatically generate dynamic Flash charts to be embedded in web applications. It is noteworthy that FusionCharts pioneered the use of Adobe Flash for statistics charting [22].

In our application, the management of data is of vital importance, since we need to efficiently access a very large quantity of data. Thus we need to use a database management system (DBMS), such as Oracle MySQL, Microsoft SQL Server, or PostgreSQL, which has been our selected DBMS. PostgreSQL is open-source software, which is not controlled by any single company. PostgreSQL is a fairly powerful DBMS, offering unlimited database size, several types of indexes, and rich database capabilities.

### Difficulties

During the deployment of the application, we faced several difficulties respecting raw data access. Specifically, we did not have real-time access to the ADVIA CentraLink database or to the Linemaster database, since the server was in a different subnet. The Linemaster problem was resolved by deploying a virtual private network to gain access to the Linemaster database. To do this, we installed and configured a VNP server on an intermediate machine, with access to both Linemaster and the server where our application is deployed, and a client in our server. We used OpenVPN for the VPN implementation [23].

The ADVIA CentraLink system problem was more difficult to solve. This system offers the possibility of exporting its database into CSV files, which in turn can be parsed by our application to retrieve the data, albeit indirectly. Until recently, the only way to trigger this data exportation was by hand, which meant that the exportation was not schedulable. Nevertheless, an AutoHotkey script was developed to simulate the movements of the mouse and keystrokes of the keyboard. AutoHotkey is a free and open-source automation software frequently used to simulate the actions of the user with the keyboard and mouse. A recent upgrade of the ADVIA CentraLink system client enables the creation of scheduled data exports; as a consequence, the AutoHotkey scripts are no longer needed.

**Figure 4:**
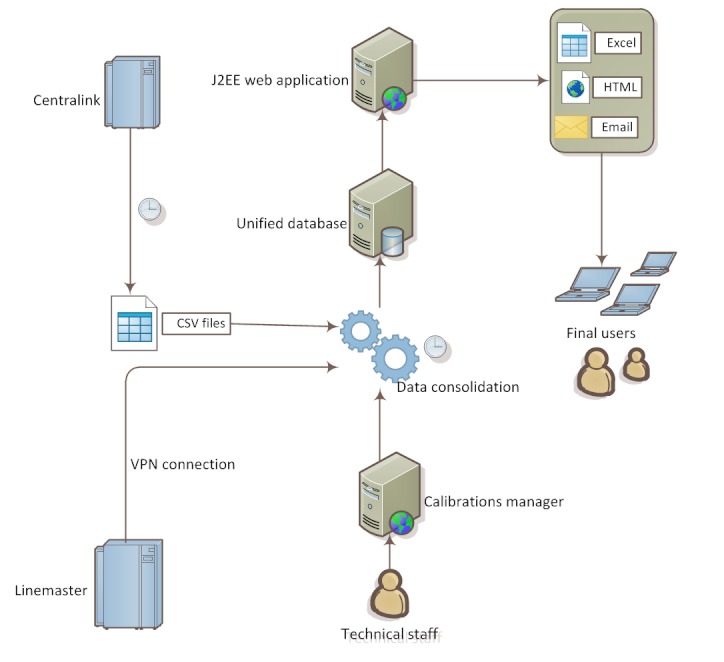
System deployment.

A further difficulty was the synchronization of the clocks of the components (the server where our application runs, the ADVIA CentraLink system and Linemaster server), in order to calculate meaningful turn-around times that involve timestamps from different sources. Finally, there are data that cannot be exported by the instruments, and thus cannot be provided by the ADVIA CentraLink system, such as the calibrations, which are needed to calculate the correct number of tests. In this case, we have implemented a subsidiary web application, with which the technical staff can manually insert the data. Figure 4 shows how the application is deployed.

### Conclusions

DB4US is an application using a methodological approach that automates the processing of information related to laboratory quality indicators presented and implemented using a central data warehouse and computing ready-to-use measures. The use of this methodology for laboratory information management has a positive impact on the laboratory, improving the usage of time as well as other laboratory resources.

The introduction of this methodology has allowed access to information that otherwise would be hidden among the vast quantity of data stored in the instruments. This gives, in some cases, the key to optimize some aspects of the laboratory performance and, in all cases, access to arbitrarily complex ad-hoc indicators in a reduced amount of time, benefiting from the advantages of Internet technologies and web-based interfaces. Furthermore, the system can be used to optimize processes and reduce costs by discovering anomalous behaviors.

ADVIA, LabCell, ADVIA Centaur, IMMULIE, ADVIA CentraLink, and all associated names, are trademarked by Siemens Healthcare Diagnostics Inc. All other brands are the property of their respective owners.
